# Heme in Cardiovascular Diseases: A Ubiquitous Dangerous Molecule Worthy of Vigilance

**DOI:** 10.3389/fcell.2021.781839

**Published:** 2022-01-19

**Authors:** Yuyang Guo, Hengli Zhao, Zhibin Lin, Taochun Ye, Dingli Xu, Qingchun Zeng

**Affiliations:** ^1^ State Key Laboratory of Organ Failure Research, Department of Cardiology, Nanfang Hospital, Southern Medical University, Guangzhou, China; ^2^ Guangdong Provincial Key Laboratory of Shock and Microcirculation, Southern Medical University, Guangzhou, China; ^3^ Bioland Laboratory (Guangzhou Regenerative Medicine and Health Guangdong Laboratory), Guangzhou, China; ^4^ Department of Cardiopulmonary Rehabilitation, First Affiliated Hospital of Guangzhou University of Chinese Medicine, Guangzhou, China

**Keywords:** heme, cardiovascular diseases, atherosclerosis, aortic valve stenosis, heart failure, ferroptosis, iron overload

## Abstract

Heme, the protoporphyrin IX iron complex is widely present in the human body and it is involved in oxygen storage, electron transfer, and enzymatic reactions. However, free heme can be toxic as it catalyzes the production of reactive oxygen species, oxidizes lipids and proteins, and causes DNA damage, thereby inducing a pro-inflammatory environment. The generation, metabolism, and degradation of heme in the human body are regulated by precise mechanisms to ensure that heme remains non-toxic. However, in several types of cardiovascular diseases, impaired metabolism and exposure to heme may occur in pathological processes, including neovascularization, internal hemorrhage, ischemia, and reperfusion. Based on years of research, in this review, we aimed to summarize the underlying mechanisms by which heme contributes to the development of cardiovascular diseases through oxidative stress, relative pathway gene expression regulation and phenotypic changes in cells. Excess heme plays a detrimental role in atherosclerosis, heart failure, myocardial ischemia-reperfusion injury, degenerative aortic valve stenosis, cardiac iron overload. Recent researches revealed that in some cases heme involved in cardiac damage though ferroptosis. Thus, heme concentrations beyond normal levels are dangerous. Further research on the role of heme in cardiovascular diseases is needed.

## 1 Introduction

Heme, chemically named as protoporphyrin IX iron complex, comprises an iron atom that is bound to heterocyclic tetrapyrrole porphyrin ring (Sawicki et al., 2015a). It is mainly involved in oxygen storage, transfer, and activation and electron transfer in the form of hemoglobin, myoglobin, cytochrome P450, and cytochrome c (Shimizu et al., 2019). The ability of heme to perform redox reactions is attributed to its iron atom. Iron, a d-block transition metal, easily donates and accepts electrons (Pantopoulos et al., 2012). In addition, proteins that contain heme can form different signaling molecules, enzymes and hormones (Derbyshire and Marletta, 2012).

### 1.1 Heme Biosynthesis

**FIGURE 1:**
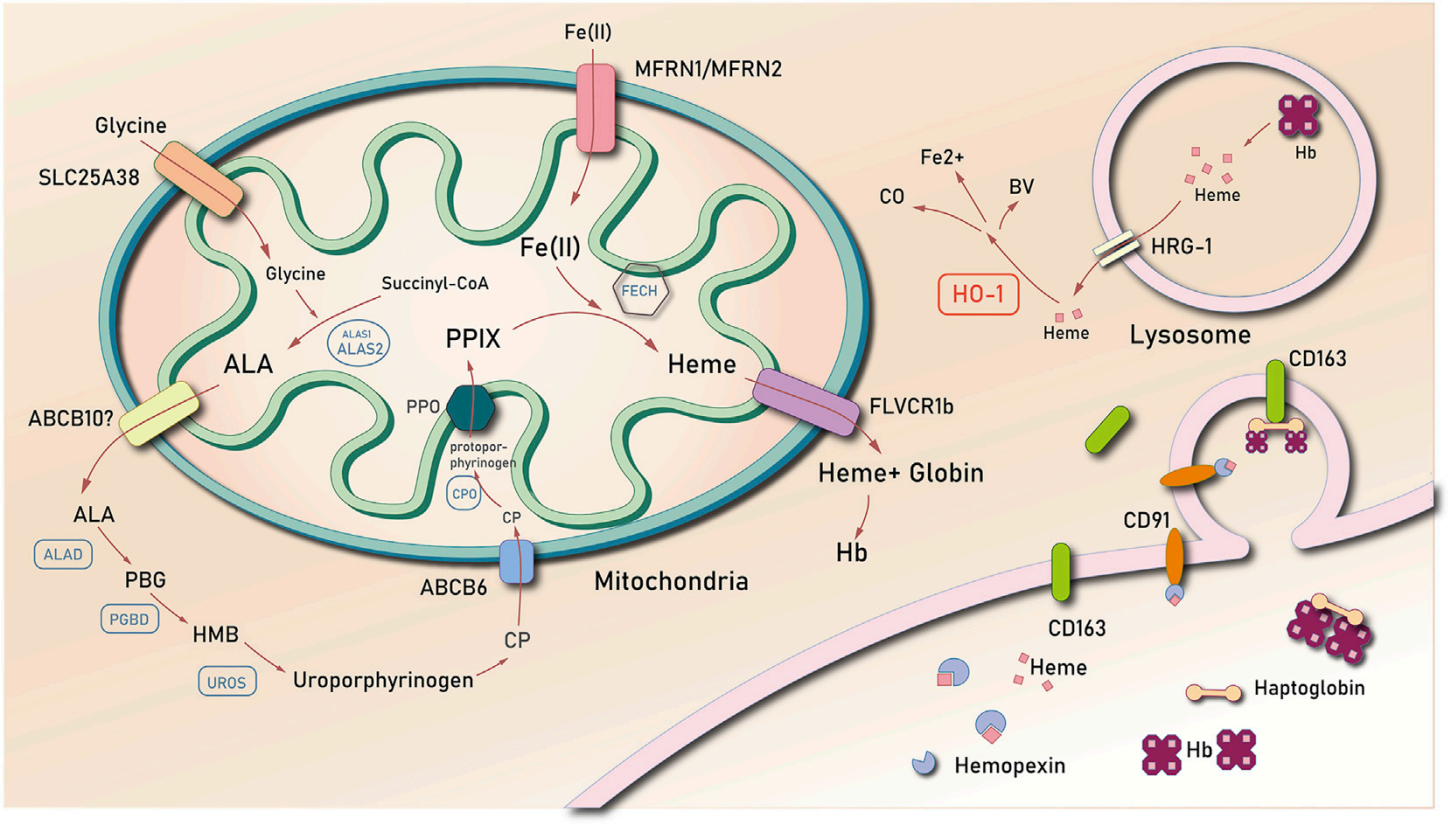
The biosynthesis and degradation of heme. Abbreviations: δ-aminolevulinic acid (ALA); coproporphyrinogen III (CP); protoporphyrin IX (PPIX); ferrochelatase (FECH); feline leukemia virus subgroup C cellular receptor (FLVCR); heme oxygenase 1 (HO-1); hemopexin (Hx); low-density lipoprotein receptor-related protein/CD91 (LDR/CD91); heme-responsive gene 1 (HRG-1); ATP binding cassette subfamily B member 6 (ABCB6); ATP binding cassette subfamily B member 10 (ABCB10); Mitoferrin-1 (Mfrn1); ALA dehydrase (ALAD); uroporphyrinogen III synthase (UROS); hydroxy-methyl bilane (HMB); porphobilinogen (PBG); PBG deaminase (PGBD); hemoglobin (Hb).

Heme is mainly generated in the mitochondria. It is formed by a series of reactions that starts with the condensation of succinyl coenzyme A and glycine, which results in the formation of the compound δ-aminolevulinic acid (ALA). This reaction is mediated by the rate-limiting enzyme, ALA synthase (ALAS) (Bonkovsky et al., 2013). ALA is transported from the mitochondria to the cytoplasm, where it is converted into coproporphyrinogen III (CP) through additional enzymatic reactions. It is then imported back to the mitochondria, in which CP forms protoporphyrin IX (PPIX). Iron is then inserted into PPIX by ferrochelatase (FECH). The newly generated heme may directly be used in the formation of respiratory chain proteins, or it may be transported into the cytosol *via* the feline leukemia virus subgroup C cellular receptor (FLVCR) (Chiabrando et al., 2012) for the formation of hemoglobin (Muckenthaler et al., 2017) (Figure 1).

### 1.2 Heme Degradation

Heme is predominantly concentrated in the red blood cells (RBCs) of mammals. This implies that there is a higher proportion of heme degradation, which results from the liberation of hemoglobin from senescent erythrocytes. Heme is catabolized by the macrophages of the reticuloendothelial system (de Back et al., 2014). In most cases, senescent RBCs are phagocytosed by specialized macrophages in the spleen, liver, and bone marrow (de Back et al., 2014). Phagosomes carrying RBCs combine with lysosomal vesicles to form erythrocyte-containing lysosomes, wherein RBCs are degraded to release heme. Heme is then exported into the cytosol *via* the lysosome heme transporter, HRG1 (Rajagopal et al., 2008; Yuan et al., 2012). Notably, free heme is released into the cytoplasm, which when present in high concentrations, causes downregulation of Bach1, a mammalian heme-responsive transcription factor that suppresses the activation of the heme oxygenase 1 (HO-1) gene (Sassa, 2004). There are at least two functionally active heme oxygenase isozymes: HO-1 and HO-2. The former is inducible and is the first and rate-limiting enzyme of the heme degradation pathway, whereas the latter is relatively constitutive.

Heme is degraded into ferrous iron and equimolar amounts of biliverdin-IX-α and carbon monoxide. This reaction is catalyzed by heme oxygenase (Ponka et al., 2017). HO-1 catalyzes the cleavage of the alpha-methylene linked carbon on the heme molecule to release the products. The body therefore maintains free heme concentrations at an extremely low level that is undetectable in the plasma of healthy individuals. Furthermore, the concentration of labile hemin is maintained to as low as 20–40 nM in the cytosol of *Saccharomyces cerevisiae* (Hanna et al., 2018) and to even lower concentrations in the mitochondria and nucleus (<2.5 nM) (Hanna et al., 2016).

During intravascular hemolysis, macrophages phagocytose free heme. This process is mediated by hemopexin (Hx), a circulating latent plasma carrier protein mainly synthesized in the liver with an extremely high affinity to heme (Nielsen et al., 2010). As a heme scavenger, Hx binds with heme, which results in the increased solubility of heme. The soluble heme diminishes its oxidative activity (Eskew et al., 1999). The Hx-heme complex combines with low-density lipoprotein receptor-related protein/CD91 (LDR/CD91), a transmembrane protein expressed in many types of cells, including macrophages, fibroblasts, hepatocytes and adipocytes, adsorbing the Hx-heme complex from the circulation (Moestrup et al., 1992; Hvidberg et al., 2005) and mediating endocytosis of the complex. When the Hx-Heme complex is degraded in the lysosome, LRP/CD91 dissociates from the complex and is then recycled to the cell membrane. LRP/CD91 might be the key protein to import the Hx-heme complex since the heme-induced increasement of HO-1 mRNA transcription was LRP/CD91-dependent (Hvidberg et al., 2005).

### 1.3 Heme Transporters

There have been several studies on heme transmembrane transport, which have resulted in the discovery of a number of potential heme transporters. In 2005, Shayeghi et al. (2005) identified a transmembrane protein called SLC46A1, a heme carrier protein 1 (HCP1). HCP1 is highly localized in the apical membrane of duodenal enterocytes and is found to promote heme absorption in the intestines (Shayeghi et al., 2005; Chung et al., 2012). The intracellular location of HCP1 makes it sensitive to changes in iron stores. Most HCP1 proteins are localized in the membranes of the brush borders of duodenal enterocytes under iron-deficient conditions. Contrastingly, HCP1 proteins retreat into the cytoplasm in iron-loaded circumstances (Shayeghi et al., 2005). However, Qiu et al. (2006) demonstrated that HCP1, which is previously thought to carry heme, is a proton-coupled folate transporter (PCFT) (Qiu et al., 2006; Ponka et al., 2017). According to the results of the K_m_ test performed by Qiu et al. (2006), the affinity of methyl tetrahydrofolate to SLC46A1 is higher than that of heme (Qiu et al., 2006; Andrews, 2007). Studies have shown that in families with hereditary folate malabsorption whose SLC46A1 gene carries a deficient mutation, the affected children are required to take high doses of supplemental folate to thrive. However, these children have no apparent defect in iron metabolism. These findings implicate SLC46A1 as a folate transporter rather than a heme carrier protein (Qiu et al., 2006; Andrews, 2007).

The heme-responsive gene 1 (HRG-1) is a conserved, membrane-bound permease that binds with and translocates heme in *Caenorhabditis elegans* and humans (Yuan et al., 2012). For instance, Rajagopal et al. (2008) found that the reactive high expression of HRG-1 in a low heme environment promotes the importation of heme in *C. elegans*. The mammalian homolog of HRG-1, which is expressed in various tissues, including the brain, heart, and kidney, is also a heme transporter from the extracellular space (Sawicki et al., 2015a). Delaby et al. (2012) found that HRG-1 proteins transport heme to the cytoplasm from lysosome. HRG-1 proteins are recruited to the erythrophagosome during erythrophagocytosis, while heme is derived from the digestion of ingested hemoproteins in the lysosome (Rajagopal et al., 2008; Yuan et al., 2012).

Feline leukemia virus subgroup C receptor (FLCRV1/2), a member of the SLC49 family, is a cell surface transporter that is crucial for erythrocyte generation and intracellular iron homeostasis (Duffy et al., 2010; Khan and Quigley, 2013). FLCRV1 acts as a heme exporter. The dysfunction of FLVCR1 arises from a mutation that impedes the development of erythroid progenitors due to heme toxicity, thereby resulting in Diamond-Blackfan anemia (Khan and Quigley, 2013). Notably, there are two different isoforms of FLVCR1. FLVCR1a is expressed on the plasma membrane, whereas FLVCR1b is expressed only in the mitochondria. Heme is exported by extracellular heme-binding proteins, such as albumin or hemopexin (Hpx), *via* FLVCR1a. Furthermore, the heme-export efficiency of FLVCR1a is directly proportional to the affinity of the extracellular protein for heme (Yang et al., 2010). The subcellular localization and function of FLVCR1b remain elusive. Contrastingly, FLVCR2 is more likely to mediate heme uptake (Duffy et al., 2010). Mammalian cells and *Xenopus laevis* oocytes that express FLVCR2 show an increase in heme uptake and a more sensitive response to heme toxicity. Heme toxicity is decreased after silencing FLVCR2 *via* siRNA or by binding the FLVCR2 receptor with the specific inhibitor called the FY981 FeLV envelope protein (Duffy et al., 2010). These observations suggest that FLVCR2 might induced toxicity through the import of excess heme.

### 1.4 Toxicity of Heme

Heme is poorly soluble in aqueous solution (Smith, 1975). The most widely used method to solubilize heme is to dissolve it in 0.001–0.01 M sodium hydroxide (Smith, 1975) or in dimethyl sulfoxide (DMSO) (Bohle et al., 2012). The oxidation state of iron in heme complexes is diverse, and the two most common are ferric protoporphyrin IX (Fe (III) complex), which is commonly known as hemin and hematin, and ferrous protoporphyrin IX (Fe(II) complex), which is commonly known as heme. In this review, heme is used as a general term to refer to either of the iron oxidation states. Free heme can be toxic at concentrations that exceed 1 µM because it promotes the formation of reactive oxygen species (ROS) (Sassa, 2004), oxidizes lipids and proteins, damages DNA (Tappel, 1953; Aft and Mueller, 1983; Aft and Mueller, 1984; Vincent, 1989; Chiabrando et al., 2014; Roumenina et al., 2016), and induces pro-inflammatory reactions in various cells. It is also suggested that heme catalyzes the production of hydroxyl radicals through the Fenton reaction in the same way as that of free Fe^2+^ (Sadrzadeh et al., 1984). However, based on the ESR spin trapping technique, Schmitt et al. (1993) found that heme likely reacts with lipids and alkanes rather than with H_2_O_2_ and hydroxyl radicals. However, the hypothesis of the heme-induced Fenton reaction remains contradictory. In aqueous buffers under physiological conditions, heme forms a stable and inert oxygen-bridged dimer through an Fe-O-Fe linkage (Brown et al., 1969; Silver and Lukas, 1983). Further research supports that heme causes cellular oxidative damage by embedding in the phospholipid biological membrane and by inducing the catalytic oxidation of organic compounds (Liu et al., 1985; Schmitt et al., 1993). This review further discusses the mechanism by which heme affects cardiovascular diseases.

While heme causes cytotoxicity in various ways, it is a strong inducer of heme oxygenase-1 (HO-1), a protein generally considered cytoprotective against oxidative injury and other cellular stresses in various disease settings (Ortiz de Montellano, 2000). Carbon monoxide (CO) and biliverdin (BV) are the by-products of heme degradation *via* heme oxygenase. They control cellular processes such as inflammation, apoptosis, and antioxidant defense (Aft and Mueller, 1983; Stocker et al., 1987; Jansen et al., 2010; Jansen and Daiber, 2012; Almeida et al., 2015). For instance, HO-1 and its by-products play a protective role against the progression of atherosclerosis through the inhibition of nuclear factor kappa-light-chain-enhancer of activated B cells (NF-κB activation), which results in the attenuation of tumor necrosis factor (TNF)-α-induced upregulation of vascular cell adhesion molecule 1 (VCAM-1) and E-selectin in endothelial cells (ECs) and in the decreased expression of TNF-α, interleukin (IL)-1β, and macrophage inflammatory protein-1β in macrophages (Wu et al., 2011; Barbagallo et al., 2013; Cheng et al., 2014; Fredenburgh et al., 2015). Simultaneously, Fe2+ generated from heme degradation increases the intracellular labile iron pool (LIP), thus leading to iron-catalyzed ROS (Kakhlon and Cabantchik, 2002; Yuan et al., 2004; Lane et al., 2015). In response to increasing LIP, cells rapidly express ferritin, an intracellular iron storage protein with ferroxidase activity and iron sequestration capability (Clegg et al., 1980; Lane et al., 2015; Hagen et al., 2017). Therefore, in various diseases and in different research models, determining whether heme is harmful or protective is complicated. Its harmful and protective effects may be closely related to its concentration, time, and the environmental conditions in which it is exposed to. The protective effects of HO-1 and heme metabolites are not discussed in detail in this review.

## 2 HEME AND ATHEROSCLEROSIS

### 2.1 Heme Accumulation in Atherosclerosis

The atherosclerotic plaques, especially the necrotic core of unstable plaques, contain apoptotic macrophages, erythrocytes and its metabolites (heme and hemoglobin), and cytotoxic substances (cholesterol crystals, cholesterol esters, oxidized lipids, fibrin, inorganic mineral-like hydroxyapatite, iron, and calcium) (Li et al., 2006). In the early 80 s, studies have demonstrated that there is a complicated microvascular network that has extended from the adventitia into the thickened intima. These neovessels have incomplete endothelial lining and have no adhesion and support of smooth muscle cells. They are too weak to maintain their integrity, thereby leading to leaks and recurrent hemorrhages (Virmani et al., 1998). Kolodgie et al. (2003) found that plaques with cores or thin caps in the late stage of necrosis have a marked increase in glycophorin A, an erythrocyte surface antigen, and iron deposits in regions of cholesterol clefts compared with those in the early stage of necrosis. It has also been suggested that RBCs and their related metabolites enter the plaques through fissures on the surface of atherosclerotic lesions (Nagy et al., 2010). According to the histological examination of atherosclerotic carotid lesions collected from patients undergoing carotid endarterectomy, Hellings et al. (2010) demonstrated that an increase in neovascularization and intraplaque hemorrhage is correlated with adverse clinical cardiovascular outcomes. However, this increase is independent of other clinical risk factors and medications, such as previous vascular intervention, bilateral carotid stenosis, hypertension, statin and dipyridamole use, and C-reactive protein and high-density lipoprotein levels (Hellings et al., 2010). Intraplaque neovascularization and intraplaque hemorrhages are crucial processes in the conversion of plaques from stable to unstable. Numerous studies have shown that RBCs, RBC membranes, hemoglobin, heme, iron, and other RBC products that enter the plaque contribute to plaque inflammation and toxicity (Nagy et al., 2010). In this review, we elaborate the relevant mechanisms induced by heme.

The prosthetic group of heme is tightly bound to hemoglobin. This bond is weakened in oxidized forms of hemoglobin (Hrkal et al., 1974). Both methemoglobin (metHb, HbFe3+αβ) and ferryl hemoglobin (HbFe4+αβ) release heme, which is captured by albumin, α1-microglobulin, and lipoproteins such as low-density lipoprotein (LDL) and high-density lipoprotein (HDL) in the absence of the plasma heme scavenger Hx (Chistiakov et al., 2015). When exposed to oxidized atherosclerotic plaque materials, erythrocytes are lysed; the liberated hemoglobin is oxidized; and heme dissociates from the oxidized hemoglobin (Nagy et al., 2010).

### 2.2 Impact of Heme in Atherosclerosis

Heme is a well-known pro-oxidant (Sadrzadeh et al., 1984). The lipophilic properties of heme enable its combination with low reactive organics (ROOH), facilitate the production of large amounts of highly reactive alkoxyl (RO•) and peroxyl (ROO•) radicals (Van der Zee et al., 1996), and ultimately stimulate the autocatalytic lipid peroxidation cascades, which have a key role in the formation of atherosclerotic lesions. Aside from increasing the production of oxidized LDLs (ox-LDLs), heme also stimulates nicotinamide adenine dinucleotide phosphate oxidase (NADPHox)-dependent ROS generation in a protein kinase C (PKC)-dependent manner in a variety of cell types (Arruda et al., 2006; Moraes et al., 2012). In neutrophils, NADPHox-derived ROS upregulate NF-κB activity and B-cell lymphoma-extra large [Bcl-X (L)] accumulation and inhibit Bax insertion into the mitochondria, thus resulting in pro-inflammatory and anti-apoptotic responses to neutrophils (Moraes et al., 2012).

In vascular smooth muscle cells (VSMCs), NADPHox-derived ROS activate redox-sensitive signaling pathways, such as ERK-2, leading to free-heme-induced concentration-dependent migration and proliferation of VSMCs (Moraes et al., 2012). However, several contrasting results suggest that heme-derived HO-1 overexpression and heme decomposition products, including carbon monoxide (CO) and biliverdin, can suppress the proliferation of VSMCs, especially when heme concentration is maintained *in vitro* at a low level (5 μM) for 7–21 days (Chang et al., 2008). Gáll et al. (2018) found that heme triggers ER stress in a time- and dose-dependent manner in human aortic smooth muscle cells (HAoSMCs) *via* all three endoplasmic reticulum (ER)-related classical pathways, which include activating transcription factor (ATF)4 expression, X Box Binding Protein 1 (XBP1) mRNA splicing, and ATF6 cleavage.

The effects of heme on endothelial cells have also been explored. In the 1990s, Balla et al. carried out many essential studies on the toxicity of heme to vascular endothelial cells. They found that heme exposure synergistically amplified the cytotoxic effects of oxidants in endothelial cells, exacerbating their damage by polymorphonuclear leukocytes, a kind of cells that tended to marginate along endothelial surfaces in the presence of inflammatory mediators (Balla et al., 1991a). Free heme released from ferri (FeIII)hemoglobin (also named methemoglobin) might involve in atherogenesis by spontaneous inserting into LDL particles and promoting oxidation of low-density lipoprotein (LDL) into cytotoxic oxidized products to endothelial cells (Balla et al., 1991b; Balla et al., 1995; Jeney et al., 2002). Substances that strengthened heme-globin liganding, like haptoglobin or cyanide could prevent heme release from ferrihemoglobin were proved to reduce the generation of oxidized LDL (Jeney et al., 2002). Interestingly, Balla et al. proved that ferrihemoglobin, but not ferro (FeII)hemoglobin, could increase the susceptibility of LDL to oxidative modification and amplify the cytotoxic effects of oxidants in endothelial cells (Balla et al., 2000), which was probably because ferrihemoglobin released its heme moieties much more readily than ferrohemoglobin according to Bunn and Jandl’s research in 1968 (Bunn and Jandl, 1968). Instead, LDL-associated lipid hydroperoxides was able to convert ferro hemoglobin to ferrihemoglobin in a dose-dependent manner (Nagy et al., 2005). Ferryl (FeIII/FeIV = O)hemoglobin, a kind of short-lived hemoglobin forming under exposure of extracellular reactive oxygen species or lipid hydroperoxide (Giulivi and Davies, 1994), was found to accumulate in the atherosclerotic lesion (Nagy et al., 2010) and activate the inflammatory response in the resident cells of the arterial wall (Silva et al., 2009), leading to increased endothelial cell permeability and enhanced monocyte adhesion (Silva et al., 2009; Potor et al., 2013). Latest research reported that even hemoglobin-derived peptide fragments of α and β subunits had effects similar to those of ferrylhemoglobin in atherosclerotic lesions, provoking endothelial dysfunction (Posta et al., 2020). Noticeably, FerrylHb specifically induced α1-microglobulin (A1M) secretion in aortic ECs, SMCs and macrophages, which was a radical-scavenging and heme-binding protein predominantly synthesized in the liver (Berggård et al., 1997). Studies showed A1M markedly inhibited Hb oxidation and heme-driven oxidation of LDL and plaque lipids derived from atheromas (Pethő et al., 2021).

The effects of heme exposure on endothelial cells was proved time-dependent dichotomous. Brief exposure to heme about 1 h produced an endothelium susceptibility to oxidant damage, a prolonged exposure to heme for about 16 increased heme oxygenase-1 and ferritin expression for approximately 50-fold and 10-fold respectively, which stimulated high resistance of endothelial cells to oxidant-mediated injury (Balla et al., 1992; Balla et al., 1993). Increased intracellular ferritin without any increment in heme oxygenase activity were also proved to raise marked protection against oxidant challenge in endothelial cells in a dose-responsive way, which was evidently attributable to the ferroxidase activity of the heavy (H) subunit, that catalyzes the oxidation of ferrous iron to ferric iron to allow intracellular iron storage in biological systems (Lawson et al., 1989; Harrison and Arosio, 1996). Studies revealed that abundant ferritin and up-regulation of heme oxygenase-1 occurred specifically in coronary atherosclerotic, which might reflect cellular response to heme or heme-iron-generated lipid peroxidation products in the atherosclerotic lesions (Juckett et al., 1995; Wang et al., 1998). In 2001, Ishikawa et al. revealed that heme-induced (intraperitoneal injections of hemin) HO-1 expression significantly decreased atherosclerotic lesions in LDL-receptor knockout mice fed high-fat diets (Ishikawa et al., 2001). It seems heme help alleviate coronary atherosclerotic in the long run. However more researches suggested that it was oxidized LDL instead of heme itself ascribed as the cause for the induction of heme oxygenase-1 and ferritin, since when catalyzing the oxidation of LDL, heme itself underwent degradation (Nath et al., 1995; Agarwal et al., 1996; Hill-Kapturczak et al., 2003). Wagener et al. (1997) found that the expression levels of ICAM-1, VCAM-1, and E-selectin proteins in vascular endothelial cells are consistently upregulated by heme. Erdei et al. (2018) demonstrated that heme, instead of protoporphyrin IX, induces ROS-mediated Nod-like receptor family pyrin domain containing 3 (NLRP3) inflammasome activation in human endothelial cells, which stimulates the secretion of active IL-1β. Weibel-Palade bodies (WPBs) are specific secretory organelles of endothelial cells. They contain the von Willebrand factor (VWF) and a variety of other proteins that are implicated in inflammation, neovascularization, and tissue recovery (Valentijn et al., 2011). Research (Belcher et al., 2014) involving transgenic sickle cell disease mice has shown that heme-mediated nicotinamide adenine dinucleotide phosphate hydrogen (NADPH) oxidase, protein kinase C (PKC), and oxidants activate endothelial toll-like receptor 4 (TLR4)/NF-κB signaling and trigger vaso-occlusion through WPB degranulation and adhesion molecule expression. Hemin significantly upregulates microvascular endothelium IL-8 secretion. This upregulation is independent from the oxidant-sensitive transcription factors, NF-κB or AP-1 (Natarajan et al., 2007).

Classically, macrophages are divided into two types: M1 and M2, which are generally considered pro-inflammatory and anti-inflammatory, respectively. Except for the two classical types of M1 and M2 macrophages, multiple phenotypes of macrophages have been reported in atherosclerotic plaques, such as Mhem. Boyle et al. (2009) defined a certain macrophage phenotype that is associated with hemorrhagic intraplaque hemorrhage (IPH), a hemorrhage-associated macrophage population (HA-mac), which is characterized by elevated expression of CD163 and HO-1, active secretion of IL-10, and low levels of human leukocyte antigen-DR (HLA-DR). Recently, they renamed it as Mhem (Boyle et al., 2012). Mhem exists only in hemorrhagic plaques, and the elevated expression levels of CD163 and HO-1 suggests that their function is to efficiently uptake and decompose hemoglobin and heme. Perl staining results have shown the accumulation of ferritin in Mhem. In addition, results of *in vitro* experiments have indicated that IL-10 also induces CD163 expression, which may form a positive feedback loop that can polarize macrophages in the presence of the Hb/Hp complex (Boyle et al., 2009). In another study, Mhem is also referred to as M hemoglobin (Finn et al., 2012). Compared to foam cells, Mhem has no lipid retention (Finn et al., 2012) but has lower ROS levels and stronger tolerance to oxidative stress *in vitro* (Boyle et al., 2012). Moreover, the upregulation of ATF1 increases lipid transport-related protein expression, such as liver X receptor (LXR)-β, LXR-α, and ATP-binding cassette transporter (ABCA) in HA-mac, which promotes cholesterol efflux (Boyle et al., 2012). Moreover, Mhem is considered as a type of conductive cell, which eliminates hemoglobin and heme, reduces oxidative stress, and prevents macrophages from transforming into foam cells.

However, Guo et al. (2018) showed that the expression of CD163 in macrophages induces the progression of atherosclerotic plaques. Due to the increase of ferritin in Mhem and the upregulated expression of iron-export protein, Ferroportin (FPN), the content of free iron in cells decreases significantly. This leads to a decrease in iron-dependent prolyl hydroxylase domain protein 2 (PHD2) activity that leads to the activation of the hypoxia-inducible factor (HIF)1α/vascular endothelial growth factor (VEGF)-A pathway. This series of events results in endothelial VCAM expression, monocyte recruitment, angiogenesis, and vascular permeability in plaques (Guo et al., 2018). Thus far, the effect of Mhem on atherosclerosis remains contradictory.

Notably, Mhem has only been reported in atherosclerosis. Currently, the reported subtypes of macrophages in atherosclerosis include M1, M2a, M2b, M2c, Mox, MHem, and M4. Potor et al. explored the pathophysiologic role of oxidation of hemoglobin (Hb) to ferrylHb in macrophages of atherosclerosis recently. They reported that macrophages exposed to ferrylHb in atherosclerotic plaques exhibited a distinct transcriptomic profile affecting gene expressions associated with inflammation (IL-1β and TNF-α et), angiogenesis, tissue remodeling, iron metabolism, apoptosis, PI3K signaling, lipid transport, and calcification, thereby contributing to the transformation of atherosclerotic lesions toward aproatherogenic phenotype (Potor et al., 2021).

Many of the proinflammatory effects of heme have been associated with activation of TLR4 signaling in macrophages (Figueiredo et al., 2007). Amino acids W23 and Y34 on myeloid differentiation factor-2 (MD-2) has recently been identified as a heme binding site in activation of TLR4 signaling (Belcher et al., 2020). Belcher et al. reveal that heme toxicity triggers TLR4 signaling leading to endothelial cell activation and vaso-occlusion in experimental SCD mouse models, which exhibited typical signs of vascular inflammation (Belcher et al., 2003). Dutra et al. found that heme triggered the processing and secretion of IL-1β dependently on NLRP3 inflammasome, inducing lethality caused by sterile hemolysis (Dutra et al., 2014).

## 3 MYOCARDIAL DAMAGE OF HEME

In the 1990s, Bhoite-Solomon et al. (1990), Alvarado et al. (2015) discovered an association between hemin and myosin *in vitro*. Heme-injured myocytes exhibit morphological changes, reduce beating rates, and increases enzymes losses (Jeney et al., 2002). Other studies have suggested that heme modifies cardiac contractile proteins through posttranslational protein modifications by consolidating with myosin light chain 1, which results in serious contractile heart dysfunction (Alvarado et al., 2015). Heme exposure alters cardiomyocyte morphology and evokes a decrease in Ca^2+^ sensitivity (pCa50) (Bunn and Jandl, 1968), thereby causing a decrease in the contractile function of the human heart. In hemolytic mice, free heme accumulates in the heart, which drives ROS to affect Ca^2+^ homeostasis, and impairs systolic function (Ingoglia et al., 2017). *In vitro*, heme-treated adult rat cardiomyocytes have shown a significant reduction in systolic Ca^2+^ transient amplitudes (de Back et al., 2014). A possible mechanism is that heme-derived ROS may have directly modified Ca^2+^-handling proteins, such as the ryanodine receptor2 (RyR2) and the sarcoendoplasmic reticulum Ca2+-ATPase 2a (SERCA2a) (Köhler et al., 2014), and may have activated intracellular stress kinases, such as calcium/calmodulin-dependent protein kinase II (CaMKII) (Erickson et al., 2008), which phosphorylates RyR2 and exacerbates Ca^2+^ mishandling. Heme also induces contractile dysfunction in human cardiomyocytes caused by the oxidation of myofilament proteins (Alvarado et al., 2015).

These pathological processes can be instrumental in cardiac dysfunction in the form of myocardial ischemia-reperfusion injury, heart failure, and hemolytic diseases. In a murine model for hemolytic diseases, application of the heme-binding protein, hemopexin, reduces ROS generation and restores myocardial function (Vinchi et al., 2013), which suggests that heme is associated with systolic and diastolic heart dysfunction in hemolytic diseases such as sickle cell disease and thalassemia (Fukuda et al., 2001; Sachdev et al., 2007; Gladwin and Sachdev, 2012; Stoyanova et al., 2012).

Khechaduri et al. found δ-aminolevulinic acid synthase 2 (ALAS2), the rate-limiting enzyme in heme production, is upregulated in human failing hearts, leading to increased heme levels in cardiac myoblasts (Khechaduri et al., 2013). Sansbury et al. (2014) conducted a transverse aortic constriction (TAC) or permanent coronary occlusion (MI) in male C57BL/6J mice and has found that after 1 week of TAC and 5 days after MI, a significant increase in ascorbate, heme, and other indices of oxidative stress are observed in a metabolomic analysis of infarcted mouse hearts. Further studies have shown that due to heme-induced mitochondrial oxidative stress, the present ALAS2 transgenic mice has exhibited an increase in heme accumulation, aggravation of cell death, and exacerbation of cardiac dysfunction after coronary ligation compared with the control littermates (Sawicki et al., 2015b). In addition, the knockdown of ALAS2 in cultured cardiomyoblasts exposed to hypoxia has reversed the increase in heme production and cell death (Fredenburgh et al., 2015).

## 4 HEME AND DEGENERATIVE AORTIC VALVE STENOSIS

Aortic valve stenosis is characterized by a slowly progressive fibrocalcific remodeling of the valve leaflets (Rajamannan et al., 2011; Lindman et al., 2016). In the early stage of the disease, known as aortic sclerosis (AS), the valve becomes thickened and mildly calcified. Nevertheless, there is no obstruction of blood flow. Over the years, the disease develops into severe valve calcification with abnormal leaflet motion, which severely obstructs blood flow. This corresponds to calcified aortic valve stenosis (Rajamannan et al., 2007; Lindman et al., 2016). Numerous studies have shown that DAVS and atherosclerotic disease share a similar pathophysiology, which includes lipid oxidation, angiogenesis, and internal hemorrhage (Zhong et al., 2019; de Oliveira Sá et al., 2020). Aortic valve leaflets obtained from aortic valve replacement in patients with DAVS, often revealed intraleaflet hemorrhage in the valve leaflets of AS. The area of intraleaflet hemorrhage is positively related to the depth of 4-Hydroxynonenal (4-HNE) staining, which suggests oxidative tissue damage that is conducive for the development of DAVS. Oxidation of LDL in the stenotic valve may also be aggravated by the presence of hemoglobin, heme, and iron (Olsson et al., 1999; Akahori et al., 2011). In addition to intraleaflet hemorrhage, another form of intravalvular hematoma has been discovered. Morvan et al. (2019) revealed that intraleaflet iron accumulation precedes calcium deposition. Furthermore, RBCs enter the fibrosa secondary to endothelial microfissuring. The contact of valvular interstitial cells (VICs) with RBCs contributes to the osteoblastic phenotype and systematic inflammation, which are manifestations of upregulated IL-6, IL-1β, osteoprotegerin, bone sialoprotein, NF-κB, bone morphogenetic proteins (BMP)2, and muscle segment homeobox 2 (Morvan et al., 2019). However, a recent study suggests that heme-mediated activation of the Nrf2/HO-1 pathway inhibits the calcification of VICs *in vitro*, a contradiction to an earlier report (Balogh et al., 2021).

## 5 CARDIAC IRON OVERLOAD

Thalassemia, sickle cell anemia, primary hemochromatosis, and other diseases that require long-term repeated blood transfusions or iron supplements can lead to cardiac iron overload (Murphy and Oudit, 2010; Vaziri, 2012; Powell et al., 2016). Primary hemochromatosis is caused by mutations in genes that encode iron-transporting and iron-sensing proteins that disrupt the production of hepcidin. In the absence of the suppressive action of hepcidin, free iron in circulation continues to accumulate and eventually reaches the binding capacity of transferrin and ferritin. These result in the appearance of non-transferrin bound iron (NTBI) or free iron. In thalassemia and sickle cell anemia, a large number of RBCs and their metabolites in circulation are degraded by the liver and spleen. The degradative process releases excessive iron in the form of NTBI to circulation. NTBI is an unstable combination of excessive iron ions, various small molecules, such as citrate and acetate, and albumins in circulation (Grootveld et al., 1989). Therefore, the main cause of the decline in cardiac function in these patients is NTBI rather than hemoglobin. Fe^2+^ from NTBI is absorbed into cardiomyocytes through bivalent transporters, such as L-type Ca channels, ZIP8, and ZIP14 in the heart, thus inducing oxidative stress (Liuzzi et al., 2006; Kumfu et al., 2012; Wang et al., 2012; Kumfu et al., 2016). ROS production leads to suppressed SERCA2 function and contractive dysfunction. Lipid peroxidation also attacks the mitochondrial membrane, thereby leading to energy depletion, mitochondrial DNA damage and dysfunction (Das et al., 2015; Zhabyeyev and Oudit, 2017; Gordan et al., 2018). Theoretically, excessive intracellular Fe^2+^ may catalyze the cascade of lipid oxidation, which leads to ferroptosis (Shayeghi et al., 2005). However, there is still no precise evidence on the role of ferroptosis in cardiac iron overload.

A compared normo-ferremic ApoE−/− mice with iron-loaded ApoE−/− FPNwt/C326S mice reported that atherosclerosis can be exacerbated by iron-induced alterations in lipids, vascular permeabilization, sustained endothelial activation, elevated pro-atherogenic inflammatory mediators, and reduced nitric oxide availability. *In vitro*, NTBI induces ROS production and apoptosis in cultured vascular cells and stimulates MCP-1-mediated monocyte recruitment, which contributes to atherosclerosis (Vinchi et al., 2020). However, it has been reported that patients with hereditary hemochromatosis have a lower prevalence of cardiovascular disease (Demetz et al., 2020). This is because the deficiency of the haemochromatosis gene HFE increases LDL receptor expression in hepatocytes so that Kupffer cells are able to transfer more LDL-derived cholesterol to hepatocytes from circulation. Therefore, patients with hemochromatosis have a lower level of LDL-C and are less likely to develop atherosclerosis (Demetz et al., 2020).

Gbotosho et al. recently suggested that heme induced IL-6 expression and cardiac hypertrophy in patients and mice with sickle cell disease (SCD) (Gbotosho et al., 2020). Heme-laden erythrocyte membrane microparticles triggered rapid vaso-occlusions in kidneys and compromised microvascular dilation (Camus et al., 2015). Patrolling monocytes, which normally scavenge damaged cells and debris from the vasculature, express higher levels HO-1, protected SCD vasculature from vaso-occlusion (Liu et al., 2018). Although Hmox1 is usually regarded as a protective protein, Menon et al. revealed that increased heme in SCD causes upregulation of Hmox1 which consequently drives cardiomyopathy through ferroptosis (Menon et al., 2021).

## 6 Heme-related ferroptosis in cardiovascular diseases

**FIGURE 2:**
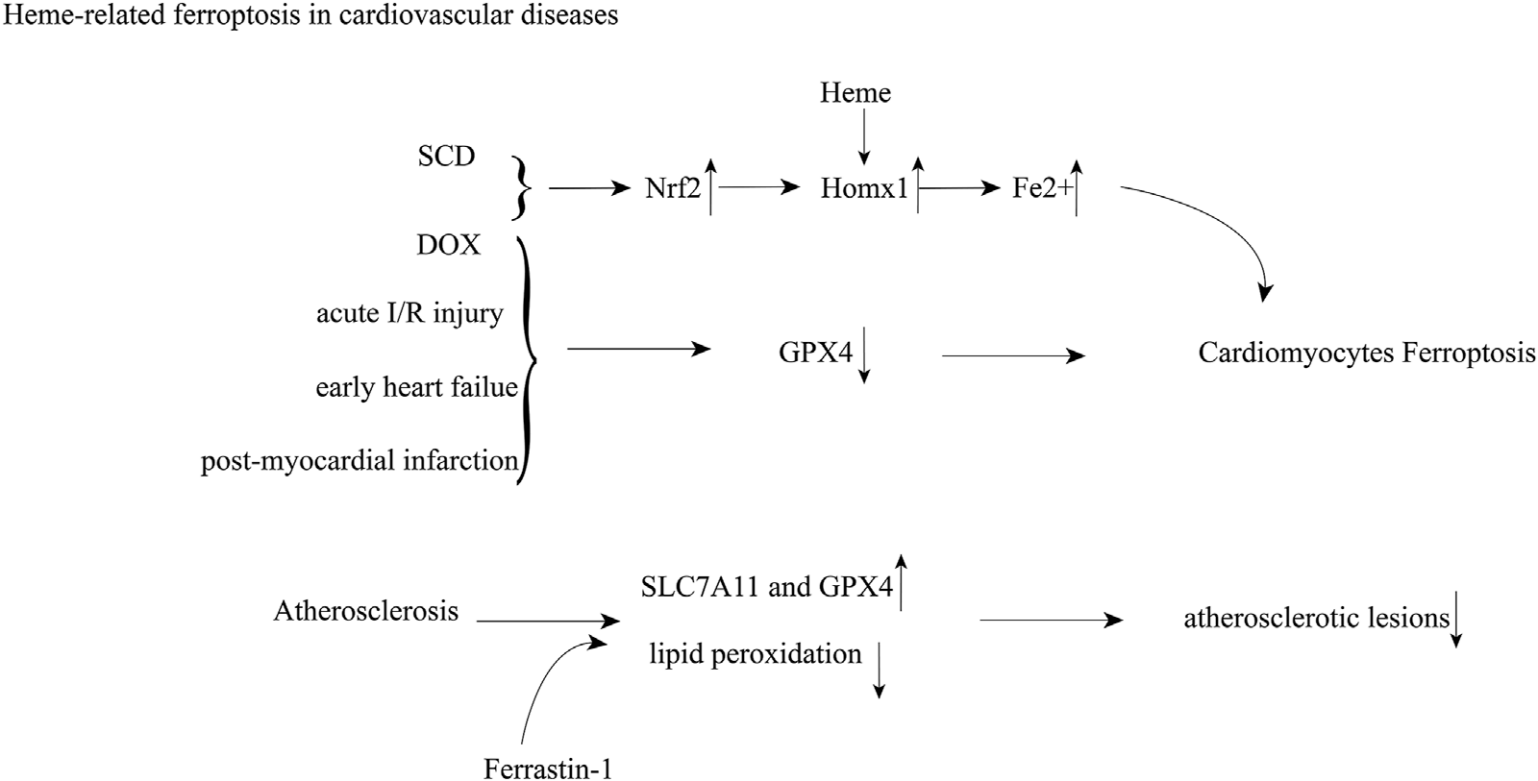
Heme-induced cardiovascular damage through ferroptosis; Abbreviations: sickle cell disease (SCD); doxorubicin (DOX); nuclear factor erythroid 2-related factor 2 (Nrf2); heme oxygenase 1 (Homx1); glutathione peroxidase 4 (GPX4); ischemia-reperfusion (I/R).

Ferroptosis, an iron-dependent form of nonapoptotic cell death known as glutathione depletion and the amplification of lipid peroxidation, was first identified in 2012 (Dixon et al., 2012). To date, it has been reported in several cardiac pathologies, including doxorubicin-induced cardiomyopathy, acute I/R injury (Fang et al., 2019; Feng et al., 2019), post-myocardial infarction, heart failure (Park et al., 2019), atherosclerosis (Bai et al., 2020) and septic heart injury (Wang et al., 2020) (Figure 2). In acute I/R injury, post-myocardial infarction, early-stage heart failure, the presence of residual myocardial iron in the post-infarcted area, and GPX4 activity decrease, which results in ferroptosis in cardiac myocytes. Menon et al. revealed that increased heme in SCD caused upregulation of Hmox1 and free iron, which consequently drives cardiomyopathy through ferroptosis (Menon et al., 2021). Inhibition or induction of Hmox1 decreased or increased cardiac ferroptosis in SCD mice, respectively (Menon et al., 2021). In DOX-induced cardiomyopathy, the Nrf2/Hmox1 pathway mediates heme degradation and releases free iron, resulting in ferroptosis (Fang et al., 2019). Ferrastin-1 decreases iron content and lipid peroxidation and upregulates the levels of SLC7A11 and GPX4, which alleviates atherosclerotic lesions (Bai et al., 2020).

## 7 Conclusion

Heme is an ancient and indispensable biomolecule in aerobic organisms. It has evolved with a wide range of complex functions and metabolism in the human body (Celis and DuBois, 2019; Shimizu et al., 2019). Imbalance in heme metabolism plays an important role in cardiovascular diseases. Heme is a metabolic intermediate, of hemoglobin, red blood cells upstream in biosynthesis and carbon monoxide, biliverdin, and divalent iron downstream in the catabolism of RBC. Therefore, discussions on heme may not be comprehensive without considering these components. Heme leads to phenotypic changes of endothelial cells, macrophages, vascular smooth muscle cells and cardiomyocytes through oxidative stress, verification activation and other pathways. Most of these changes are harmful, but some studies have shown that prolonged exposure to a small amount of heme can induce antioxidant or anti-inflammatory phenotypes in cells. Heme binding proteins, like AIM and Hx, show their potential in treating heme-induced cardiovascular diseases, which may provide therapeutic opportunities to related cardiovascular diseases. The effects of heme on blood vessels, myocardium, and heart valves need to be further investigated.
